# Supplementary material for: ProteinShader: illustrative rendering of macromolecules
Source: BMC Struct Biol. 2009 Mar 30;9:19. doi: 10.1186/1472-6807-9-19 (PMC2672931; doi:10.1186/1472-6807-9-19)
Supplement: Additional file 1 — ProteinShader program without source code. This compressed file contains the complete ProteinShader program including associated libraries, but no source code. A README.txt file gives an overview of the ProteinShader distribution, and the index.html file in the help subdirectory has directions on getting started with the program as well as a set of tutorials. [file 1472-6807-9-19-S1.zip › ProteinShader-beta-0_9_4-binary/help/tutorials/tutorials.html]

ProteinShader: Tutorials


# ProteinShader Tutorials


♦ Home


♦ Download


♦ Getting Started

Choose a Link:

Installation

Running

Troubleshooting

PDB Files


♦ Tutorials

Choose a Link:

List of Proteins

Style Options

Saving Images


♦ General Controls

Choose a Link:

Menu Bar

Keyboard Shortcuts

Mouse Motion

Motion Panel

Antialiasing


♦ Cartoon Controls

Choose a Link:

Control Panels

Selection Panel

Radio Buttons

Decorations Panel

Adding Textures

Color Panel

Visibility Panel

Side Chains Panel


♦ Atom Controls

Choose a Link:

Control Panels

Selection Panel

Radio Buttons

Color Panel

Visibility Panel

Scale Panel

Tiling Panel


♦ Developers

Choose a Link:

Compiling

Running

Code Docs

Developer Links


## List of Proteins

Ribonuclease Inhibitor   
Retinol-Binding Protein   
Porin   
Potassium Channel   
Human Growth Hormone   
3-Isopropylmalate Dehydrogenase   
c-Jun Homodimer

A short summary of
style options
is given nearer to the bottom of this page, as well as some tips on
saving images as PNG or JPEG files.

Click on the name of a protein to go to its tutorial page, or
click on one of the thumbnail images below to see it full size.

|  |  |
| --- | --- |
|  | Ribonuclease Inhibitor: Alpha/Beta HorseshoeShaped Structure |

|  | Retinol-Binding Protein: Beta-BarrelStructure |

|  | Porin: Transmembrane Beta-Barrel |

|  | Potassium Channel: Transmembrane AlphHelices |

|  | Human Growth Hormone: Four Alpha-Helix Bundle |

|  | 3-Isopropylmalate Dehydrogenase: Three-Layer (Alpha-Beta-Alpha) Sandwich |

|  | c-Jun Homodimer: Leucine Zipper Protein |


## Style Options

When a protein structure is first loaded from a Protein Data Bank file,
ProteinShader displays it as a pen-and-ink style drawing using ribbons and tubes
to represent the backbone of each polypeptide chain. Alpha-helices are drawn as
tubes, while beta-strands and general loop regions are drawn as ribbons. To
emphasize regions of protein secondary structure, alpha-helices and beta-strands
are drawn with a larger tube radius or ribbon width than loops. The
Style menu
above the canvas can be used to change the cartoon to tubes only, ribbons only,
a combination of ribbons and tubes, or to an atom-style display: space filling
(spheres), balls and sticks (spheres and cylinders), or sticks (cylinders).

The appearance of cartoon or atom-style displays can be modified extensively
with the retractable control panel on the right side of the canvas. This
tutorial will use the proteins shown below to help familiarize the user with
the control panel and its interchangeable modifier panels. The
Saving Images
section at the bottom of this page gives advice on saving an image to a
file and on using the antialising control panel to improve image quality.


## Saving Images

The images on the tutorial pages were saved as PNG (Portable Network Graphics,
www.libpng.org/pub/png)
or JPEG (Joint Photographic Experts Group,
www.jpeg.org)
files by using the Export Image submenu of the
File menu
above the canvas. For the images produced by the ProteinShader program, the PNG
format usually produces the best images because it uses lossless compression.
The JPEG format, which is lossy compression, generally produces files about
four times smaller, although the exact difference depends a great deal on the
complexity of the image. However, the JPEG images sometimes have minor
artifacts such as a little splotchiness where a color is very gradually
changing shade across a surface. In general, it is a good idea to save images
in both PNG and JPEG formats, and then go with the smaller JPEG image only if
the quality is not too much different than the PNG file.

Most of the original images for the tutorial pages were saved at a size of
a little over 600 by 600 pixels, and in all cases the
Antialiasing subpanel
was used to smooth out any jagged edges. The maximum of jittering 16 times
was used, but for most images jittering 5 or 6 times is all that is needed,
and increasing the jitter beyond that does not seem to make any difference
on a monitor with 72 pixels per inch resolution. When images needed to be
reduced in size (such as the thumbnails further above), the free GIMP (GNU
Image Reduction Program,
www.gimp.org)
was used, and the minimized images were saved with cubic filtering (best
quality).

The Export Image submenu of the
File menu also has an
"Invisible Canvas PNG..." option that allows PNG files to be saved
with a transparent background. An image file with a transparent background can
be useful for displaying images on a web page where a particular background
color is in use. If the page background color is changed, an image with a
transparent background does not need to be redone to make its background
match the page. For an example look at the top of this page.

The ability to save a GIF (Graphics Interchange Format,
 en.wikipedia.org/wiki/GIF)
image has also been added to the Export Image submenu. However, for static
screenshots, GIF is usually less desirable than PNG or JPEG images because
GIF only supports a table of 256 colors, whereas PNG or JPEG support millions
of colors. The only reason for adding GIF is that a future version of the
ProteinShader program will likely use GIF to save small animations suitable
for posting on a web site (PNG and JPEG do not support animations).


---

|  |  |  |  |
| --- | --- | --- | --- |
| Hosted by |  |  |  |

Webmaster: Joe Weber,
joe.weber AT alumni.duke.edu
  
Web site last updated: December 30th, 2008
  
© 2007–2008 Joseph R. Weber
